# 4-(2-Nitro­benzene­sulfonamido)pyri­dinium trifluoro­acetate

**DOI:** 10.1107/S1600536808020825

**Published:** 2008-07-12

**Authors:** Jiang-Sheng Li, Mei-Lian Fan, Wen-Sheng Li, Wei-Dong Liu

**Affiliations:** a School of Chemical & Biological Engineering, Changsha University of Science and Technology, Changsha 410076, People’s Republic of China; b College of Chemistry and Chemical Engineering, Hunan University, Changsha, Hunan 410082, People’s Republic of China; c Hunan Research Institute of the Chemical Industry, Changsha 410007, People’s Republic of China

## Abstract

In the title compound, C_11_H_10_N_3_O_4_S^+^·C_2_F_3_O_2_^−^, the dihedral angle between the benzene ring and the pyridinium ring is 88.7 (4)°. In the crystal structure, a network of N—H⋯O, C—H⋯O and C—H⋯F hydrogen bonds links the constituent ions. One O atom of the nitro group is disordered over two positions, with site-occupancy factors of 0.57 (2) and 0.43 (2).

## Related literature

For related structures, see: Yu & Li (2007 ▶); Li *et al.* (2008 ▶).
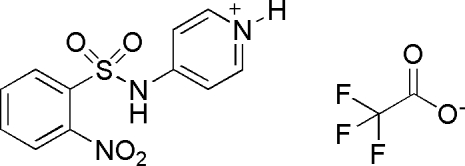


## Experimental

### 

#### Crystal data

C_11_H_10_N_3_O_4_S^+^·C_2_F_3_O_2_^−^
*M* *_r_* = 393.30
Monoclinic, 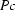

*a* = 10.666 (3) Å
*b* = 5.0619 (16) Å
*c* = 14.848 (5) Å
β = 92.823 (6)°
*V* = 800.7 (4) Å^3^
*Z* = 2
Mo *K*α radiation
μ = 0.27 mm^−1^
*T* = 294 (2) K
0.50 × 0.40 × 0.14 mm

#### Data collection

Bruker SMART 1K CCD diffractometer
Absorption correction: multi-scan (*SADABS*; Sheldrick, 1996 ▶) *T*_min_ = 0.875, *T*_max_ = 0.963
3878 measured reflections
1912 independent reflections
1351 reflections with *I* > 2σ(*I*)
*R*_int_ = 0.043

#### Refinement

*R*[*F* ^2^ > 2σ(*F* ^2^)] = 0.050
*wR*(*F* ^2^) = 0.146
*S* = 1.01
1912 reflections
252 parameters
44 restraints
H atoms treated by a mixture of independent and constrained refinement
Δρ_max_ = 0.40 e Å^−3^
Δρ_min_ = −0.28 e Å^−3^
Absolute structure: Flack (1983 ▶), 515 Friedel pairs
Flack parameter: 0.2 (2)

### 

Data collection: *SMART* (Bruker, 1997 ▶); cell refinement: *SAINT* (Bruker, 1997 ▶); data reduction: *SAINT*; program(s) used to solve structure: *SHELXS97* (Sheldrick, 2008 ▶); program(s) used to refine structure: *SHELXL97* (Sheldrick, 2008 ▶); molecular graphics: *SHELXTL* (Sheldrick, 2008 ▶); software used to prepare material for publication: *SHELXTL*.

## Supplementary Material

Crystal structure: contains datablocks global, I. DOI: 10.1107/S1600536808020825/hb2758sup1.cif

Structure factors: contains datablocks I. DOI: 10.1107/S1600536808020825/hb2758Isup2.hkl

Additional supplementary materials:  crystallographic information; 3D view; checkCIF report

## Figures and Tables

**Table 1:** Hydrogen-bond geometry (Å, °)

*D*—H⋯*A*	*D*—H	H⋯*A*	*D*⋯*A*	*D*—H⋯*A*
N2—H2⋯O6^i^	0.92 (8)	1.93 (9)	2.835 (8)	169 (7)
N1—H1*A*⋯O6	0.90 (6)	1.89 (3)	2.746 (8)	157 (7)
C3—H3⋯O1^ii^	0.93	2.41	3.310 (9)	162
C10—H10⋯F3^iii^	0.93	2.50	3.313 (12)	146

Symmetry codes: (i) 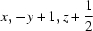; (ii) 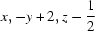; (iii) 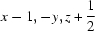.

**Fig. 1.**
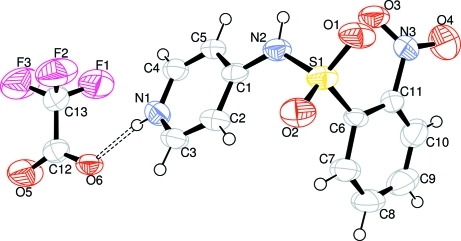
A view of (I) with displacement ellipsoids drawn at the 50% probability level (arbitrary spheres for the H atoms). Only the major disordered nitro group is shown. The dashed line indicates the H-bond.

## supplementary crystallographic information

### Comment

The title compound, (I), comprises of pyridinium cation and a trifluoroacetate anion (Fig. 1). In the cation, the short C—N distance [C1—N2 = 1.383 (9) Å] and planar conformation [C1—N2—S1 = 126.8 (5)°, C1—N2—-H2 = 114 (5)°, S1—N2—H2 = 119 (5)°] indicate that N2 possesses *sp*^2^ character despite the proximity of the strongly electron-withdrawing sulfonyl group. The benzene ring makes an angle of 88.7 (4)° with the pyridinium ring.

A network of intermolecular N—H···O, C—H···O and C—H···F hydrogen bonds (Table 1) complete the crystal packing for (I).

For related structures, see: Yu & Li (2007) and Li *et al.* (2008).

### Experimental

2-Nitro-(*N*-pyridyl)benzenesulfonamide was prepared by the method of Yu & Li (2007). Colourless blocks of (I) were grown by natural evaporation of a methanolic solution of the amide trifluoroacetate salt.

### Refinement

The N-bound H atoms were located in a difference map and refined with the restraint N—H = 0.90 (1)Å. and the C-bound H atoms were positioned geometrically (C—H = 0.93 Å) and refined as riding atoms. The constraint Uĩso~(H) = 1.2 U~eq~(C and N) was applied.

The C—F distances in the anion were restrained to 1.36 (1) Å and the N3—O4(O4') distances to 1.20 (1) Å.  The displacement parameters for the F atoms and O4/O4' were restrained to approximate to isotropic behaviour. The nitro group is partially disordered over two positions in a 0.57 (2):0.43 (2) ratio.

### Crystal data

**Table:**

C_11_H_10_N_3_O_4_S^+^·C_2_F_3_O_2_^–^	*F*_000_ = 400
*M**_r_* = 393.30	*D*_x_ = 1.631 Mg m^−^^3^
Monoclinic, *P**c*	Mo *K*α radiation λ = 0.71073 Å
Hall symbol: P -2yc	Cell parameters from 903 reflections
*a* = 10.666 (3) Å	θ = 2.8–21.0º
*b* = 5.0619 (16) Å	µ = 0.27 mm^−^^1^
*c* = 14.848 (5) Å	*T* = 294 (2) K
β = 92.823 (6)º	Block, colourless
*V* = 800.7 (4) Å^3^	0.50 × 0.40 × 0.14 mm
*Z* = 2	

### Data collection

**Table:**

Bruker SMART 1K CCD diffractometer	1912 independent reflections
Radiation source: fine-focus sealed tube	1351 reflections with *I* > 2σ(*I*)
Monochromator: graphite	*R*_int_ = 0.043
*T* = 294(2) K	θ_max_ = 25.0º
ω scans	θ_min_ = 1.9º
Absorption correction: multi-scan(SADABS; Sheldrick, 1996)	*h* = −6→12
*T*_min_ = 0.875, *T*_max_ = 0.963	*k* = −6→6
3878 measured reflections	*l* = −17→17

### Refinement

**Table:**

Refinement on *F*^2^	Hydrogen site location: difmap and geom
Least-squares matrix: full	H atoms treated by a mixture of independent and constrained refinement
*R*[*F*^2^ > 2σ(*F*^2^)] = 0.050	*w* = 1/[σ^2^(*F*_o_^2^) + (0.0892*P*)^2^ + 0.0881*P*] where *P* = (*F*_o_^2^ + 2*F*_c_^2^)/3
*wR*(*F*^2^) = 0.146	(Δ/σ)_max_ < 0.001
*S* = 1.01	Δρ_max_ = 0.40 e Å^−^^3^
1912 reflections	Δρ_min_ = −0.28 e Å^−^^3^
252 parameters	Extinction correction: none
44 restraints	Absolute structure: Flack (1983), 515 Friedel pairs
Primary atom site location: structure-invariant direct methods	Flack parameter: 0.2 (2)
Secondary atom site location: difference Fourier map	

### Special details

**Table:**

Geometry. All e.s.d.'s (except the e.s.d. in the dihedral angle between two l.s. planes) are estimated using the full covariance matrix. The cell e.s.d.'s are taken into account individually in the estimation of e.s.d.'s in distances, angles and torsion angles; correlations between e.s.d.'s in cell parameters are only used when they are defined by crystal symmetry. An approximate (isotropic) treatment of cell e.s.d.'s is used for estimating e.s.d.'s involving l.s. planes.
Refinement. Refinement of *F*^2^ against ALL reflections. The weighted *R*-factor *wR* and goodness of fit *S* are based on *F*^2^, conventional *R*-factors *R* are based on *F*, with *F* set to zero for negative *F*^2^. The threshold expression of *F*^2^ > σ(*F*^2^) is used only for calculating *R*-factors(gt) *etc*. and is not relevant to the choice of reflections for refinement. *R*-factors based on *F*^2^ are statistically about twice as large as those based on *F*, and *R*- factors based on ALL data will be even larger.

### Fractional atomic coordinates and isotropic or equivalent isotropic displacement parameters (Å^2^)

**Table:**

	*x*	*y*	*z*	*U*_iso_*/*U*_eq_	Occ. (<1)
S1	0.1917 (2)	1.1065 (3)	0.30158 (15)	0.0475 (5)	
O1	0.2002 (5)	1.2527 (9)	0.3840 (4)	0.0618 (16)	
O2	0.1765 (5)	1.2424 (10)	0.2190 (4)	0.0606 (16)	
O3	0.2046 (5)	0.7895 (10)	0.4739 (3)	0.0585 (14)	
O4	0.0306 (13)	0.694 (4)	0.5333 (8)	0.100 (5)	0.57 (2)
O4'	0.0360 (16)	0.896 (5)	0.5277 (10)	0.092 (6)	0.43 (2)
N1	0.4391 (6)	0.4397 (12)	0.1006 (4)	0.0510 (15)	
H1A	0.461 (7)	0.307 (10)	0.064 (4)	0.061*	
N2	0.3191 (6)	0.9262 (13)	0.3012 (4)	0.0483 (17)	
H2	0.364 (8)	0.898 (14)	0.355 (6)	0.058*	
N3	0.0922 (7)	0.7742 (19)	0.4702 (4)	0.077 (2)	
C1	0.3581 (6)	0.7718 (13)	0.2310 (4)	0.0392 (17)	
C2	0.3127 (7)	0.7987 (13)	0.1424 (5)	0.0464 (19)	
H2A	0.2537	0.9280	0.1265	0.056*	
C3	0.3570 (7)	0.6291 (14)	0.0778 (5)	0.049 (2)	
H3	0.3289	0.6479	0.0179	0.059*	
C4	0.4851 (7)	0.4122 (14)	0.1858 (5)	0.0486 (17)	
H4	0.5439	0.2811	0.1999	0.058*	
C5	0.4461 (7)	0.5751 (13)	0.2515 (5)	0.0404 (17)	
H5	0.4783	0.5550	0.3104	0.048*	
C6	0.0662 (6)	0.8823 (12)	0.3040 (4)	0.0367 (17)	
C7	−0.0026 (8)	0.8267 (15)	0.2243 (5)	0.059 (2)	
H7	0.0200	0.9097	0.1716	0.071*	
C8	−0.1003 (8)	0.6585 (19)	0.2199 (6)	0.074 (3)	
H8	−0.1432	0.6266	0.1649	0.088*	
C9	−0.1370 (8)	0.533 (2)	0.2967 (7)	0.075 (3)	
H9	−0.2049	0.4181	0.2939	0.090*	
C10	−0.0735 (8)	0.5798 (19)	0.3759 (6)	0.067 (3)	
H10	−0.0977	0.4954	0.4280	0.081*	
C11	0.0259 (7)	0.7498 (16)	0.3802 (5)	0.050 (2)	
F1	0.6379 (6)	−0.0984 (15)	0.0885 (4)	0.120 (2)	
F2	0.7133 (6)	0.2337 (12)	0.0429 (5)	0.112 (2)	
F3	0.7883 (6)	−0.1131 (15)	−0.0079 (5)	0.126 (2)	
O5	0.6055 (6)	−0.1151 (14)	−0.1338 (5)	0.104 (2)	
O6	0.4859 (5)	0.1277 (10)	−0.0453 (3)	0.0544 (13)	
C12	0.5818 (7)	0.0001 (15)	−0.0642 (5)	0.0535 (19)	
C13	0.6817 (7)	−0.0063 (15)	0.0123 (5)	0.062 (2)	

### Atomic displacement parameters (Å^2^)

**Table:**

	*U*^11^	*U*^22^	*U*^33^	*U*^12^	*U*^13^	*U*^23^
S1	0.0514 (10)	0.0362 (8)	0.0533 (10)	0.0036 (10)	−0.0129 (7)	−0.0053 (10)
O1	0.075 (4)	0.041 (3)	0.067 (4)	0.003 (3)	−0.021 (3)	−0.023 (3)
O2	0.069 (4)	0.052 (3)	0.060 (4)	0.003 (3)	−0.012 (3)	0.017 (3)
O3	0.055 (4)	0.067 (3)	0.051 (3)	0.006 (3)	−0.018 (3)	−0.003 (2)
O4	0.095 (8)	0.148 (11)	0.060 (7)	−0.011 (7)	0.019 (6)	0.015 (7)
O4'	0.084 (9)	0.136 (11)	0.055 (8)	0.005 (8)	0.013 (6)	−0.020 (8)
N1	0.059 (4)	0.054 (4)	0.040 (4)	−0.007 (3)	0.001 (3)	0.001 (3)
N2	0.045 (4)	0.053 (4)	0.045 (4)	0.005 (3)	−0.011 (3)	−0.001 (3)
N3	0.055 (5)	0.138 (8)	0.037 (4)	0.010 (5)	0.000 (4)	−0.010 (4)
C1	0.040 (4)	0.036 (4)	0.041 (4)	−0.012 (3)	−0.009 (3)	0.003 (3)
C2	0.057 (5)	0.040 (4)	0.041 (4)	−0.011 (4)	−0.013 (3)	0.006 (3)
C3	0.053 (5)	0.059 (5)	0.035 (4)	−0.011 (4)	−0.010 (3)	0.004 (4)
C4	0.047 (4)	0.055 (4)	0.042 (4)	−0.006 (4)	−0.005 (3)	0.005 (3)
C5	0.040 (4)	0.043 (4)	0.038 (4)	−0.002 (3)	−0.008 (3)	0.002 (3)
C6	0.039 (4)	0.039 (4)	0.030 (4)	0.013 (3)	−0.010 (3)	−0.004 (3)
C7	0.056 (5)	0.074 (5)	0.045 (4)	−0.010 (4)	−0.010 (4)	0.005 (4)
C8	0.062 (6)	0.098 (7)	0.060 (5)	−0.022 (5)	−0.017 (4)	−0.007 (5)
C9	0.052 (5)	0.084 (6)	0.088 (7)	−0.011 (5)	−0.009 (5)	−0.006 (5)
C10	0.050 (5)	0.086 (6)	0.067 (6)	0.000 (5)	0.007 (5)	0.017 (5)
C11	0.041 (5)	0.070 (5)	0.038 (4)	0.013 (4)	−0.002 (4)	−0.002 (4)
F1	0.093 (4)	0.181 (6)	0.085 (4)	0.005 (5)	−0.022 (3)	0.034 (4)
F2	0.093 (4)	0.115 (4)	0.125 (4)	−0.013 (4)	−0.031 (3)	−0.034 (4)
F3	0.074 (4)	0.160 (5)	0.140 (5)	0.047 (4)	−0.020 (3)	−0.042 (4)
O5	0.081 (5)	0.141 (6)	0.087 (5)	0.033 (4)	−0.013 (4)	−0.066 (4)
O6	0.048 (3)	0.068 (3)	0.046 (3)	0.013 (3)	−0.012 (2)	−0.007 (2)
C12	0.050 (5)	0.051 (4)	0.058 (5)	0.002 (4)	−0.004 (4)	0.005 (4)
C13	0.054 (6)	0.067 (5)	0.065 (5)	−0.009 (5)	0.002 (4)	−0.004 (4)

### Geometric parameters (Å, °)

**Table:**

S1—O2	1.408 (6)	C4—H4	0.9300
S1—O1	1.429 (5)	C5—H5	0.9300
S1—N2	1.637 (7)	C6—C7	1.390 (9)
S1—C6	1.757 (7)	C6—C11	1.401 (10)
O3—N3	1.200 (8)	C7—C8	1.345 (11)
O4—N3	1.239 (9)	C7—H7	0.9300
O4'—N3	1.233 (10)	C8—C9	1.378 (13)
N1—C3	1.331 (9)	C8—H8	0.9300
N1—C4	1.341 (9)	C9—C10	1.349 (12)
N1—H1A	0.90 (6)	C9—H9	0.9300
N2—C1	1.383 (9)	C10—C11	1.365 (11)
N2—H2	0.92 (8)	C10—H10	0.9300
N3—C11	1.485 (10)	F1—C13	1.328 (7)
C1—C2	1.387 (9)	F2—C13	1.334 (7)
C1—C5	1.391 (10)	F3—C13	1.308 (8)
C2—C3	1.387 (10)	O5—C12	1.223 (10)
C2—H2A	0.9300	O6—C12	1.253 (9)
C3—H3	0.9300	C12—C13	1.520 (11)
C4—C5	1.358 (9)		
			
O2—S1—O1	119.5 (3)	C4—C5—C1	120.3 (7)
O2—S1—N2	109.2 (4)	C4—C5—H5	119.8
O1—S1—N2	105.9 (3)	C1—C5—H5	119.8
O2—S1—C6	106.1 (3)	C7—C6—C11	114.9 (7)
O1—S1—C6	109.5 (3)	C7—C6—S1	118.9 (5)
N2—S1—C6	105.9 (3)	C11—C6—S1	126.2 (5)
C3—N1—C4	121.3 (7)	C8—C7—C6	123.0 (7)
C3—N1—H1A	125 (5)	C8—C7—H7	118.5
C4—N1—H1A	113 (5)	C6—C7—H7	118.5
C1—N2—S1	126.8 (5)	C7—C8—C9	120.1 (8)
C1—N2—H2	114 (5)	C7—C8—H8	120.0
S1—N2—H2	119 (5)	C9—C8—H8	120.0
O3—N3—O4'	117.1 (12)	C10—C9—C8	119.4 (9)
O3—N3—O4	123.5 (10)	C10—C9—H9	120.3
O4'—N3—O4	49.2 (9)	C8—C9—H9	120.3
O3—N3—C11	118.5 (6)	C9—C10—C11	120.4 (9)
O4'—N3—C11	116.0 (11)	C9—C10—H10	119.8
O4—N3—C11	114.0 (10)	C11—C10—H10	119.8
N2—C1—C2	123.7 (7)	C10—C11—C6	122.2 (7)
N2—C1—C5	117.7 (6)	C10—C11—N3	115.4 (8)
C2—C1—C5	118.6 (7)	C6—C11—N3	122.4 (7)
C1—C2—C3	118.7 (7)	O5—C12—O6	129.9 (7)
C1—C2—H2A	120.7	O5—C12—C13	117.0 (7)
C3—C2—H2A	120.7	O6—C12—C13	113.1 (7)
N1—C3—C2	120.9 (7)	F3—C13—F1	113.4 (8)
N1—C3—H3	119.6	F3—C13—F2	104.3 (7)
C2—C3—H3	119.6	F1—C13—F2	97.1 (7)
N1—C4—C5	120.2 (7)	F3—C13—C12	115.0 (7)
N1—C4—H4	119.9	F1—C13—C12	112.4 (7)
C5—C4—H4	119.9	F2—C13—C12	113.0 (7)
			
O2—S1—N2—C1	−43.3 (7)	C6—C7—C8—C9	0.5 (14)
O1—S1—N2—C1	−173.2 (6)	C7—C8—C9—C10	−0.5 (14)
C6—S1—N2—C1	70.6 (7)	C8—C9—C10—C11	0.2 (14)
S1—N2—C1—C2	17.6 (10)	C9—C10—C11—C6	0.0 (13)
S1—N2—C1—C5	−161.0 (5)	C9—C10—C11—N3	−177.0 (8)
N2—C1—C2—C3	−178.8 (6)	C7—C6—C11—C10	−0.1 (11)
C5—C1—C2—C3	−0.2 (10)	S1—C6—C11—C10	179.5 (6)
C4—N1—C3—C2	−2.3 (10)	C7—C6—C11—N3	176.8 (7)
C1—C2—C3—N1	1.6 (10)	S1—C6—C11—N3	−3.6 (10)
C3—N1—C4—C5	1.5 (10)	O3—N3—C11—C10	140.3 (8)
N1—C4—C5—C1	0.0 (10)	O4'—N3—C11—C10	−72.6 (16)
N2—C1—C5—C4	178.1 (6)	O4—N3—C11—C10	−17.9 (15)
C2—C1—C5—C4	−0.6 (10)	O3—N3—C11—C6	−36.8 (12)
O2—S1—C6—C7	15.4 (6)	O4'—N3—C11—C6	110.3 (16)
O1—S1—C6—C7	145.6 (6)	O4—N3—C11—C6	165.0 (13)
N2—S1—C6—C7	−100.6 (6)	O5—C12—C13—F3	6.6 (11)
O2—S1—C6—C11	−164.2 (6)	O6—C12—C13—F3	−173.1 (7)
O1—S1—C6—C11	−34.0 (7)	O5—C12—C13—F1	−125.1 (9)
N2—S1—C6—C11	79.8 (7)	O6—C12—C13—F1	55.2 (9)
C11—C6—C7—C8	−0.2 (12)	O5—C12—C13—F2	126.1 (9)
S1—C6—C7—C8	−179.8 (7)	O6—C12—C13—F2	−53.5 (9)

### Hydrogen-bond geometry (Å, °)

**Table:**

*D*—H···*A*	*D*—H	H···*A*	*D*···*A*	*D*—H···*A*
N2—H2···O6^i^	0.92 (8)	1.93 (9)	2.835 (8)	169 (7)
N1—H1A···O6	0.90 (6)	1.89 (3)	2.746 (8)	157 (7)
C3—H3···O1^ii^	0.93	2.41	3.310 (9)	162
C10—H10···F3^iii^	0.93	2.50	3.313 (12)	146

Symmetry codes: (i) *x*, −*y*+1, *z*+1/2; (ii) *x*, −*y*+2, *z*−1/2; (iii) *x*−1, −*y*, *z*+1/2.
